# Power and lie detection

**DOI:** 10.1371/journal.pone.0269121

**Published:** 2022-06-09

**Authors:** Joanna Ulatowska, Aleksandra Cislak

**Affiliations:** 1 Department of Psychology, Nicolaus Copernicus University, Toruń, Poland; 2 Center for Research on Social Relations, Institute of Psychology, University of Social Sciences and Humanities, Warsaw, Poland; DePaul University, UNITED STATES

## Abstract

Social power undermines focus on others and increases reliance on stereotype-consistent information. Thus, power may enhance focus on stereotypical cues to deception, thereby decreasing lie detection accuracy. In three studies, we tested whether having power affects lie detection accuracy. Participants (overall *N* = 502) were asked to identify truthful and lying candidates (*N* = 12) during mock job interviews. Study 1 was a field experiment involving employees who held managerial and non-managerial positions (*N* = 88). In the following laboratory experiments, we manipulated power and asked participants to imagine themselves as managers (Study 2, *N* = 214) or provided them with control over resources and the ability to reward others (Study 3, *N* = 200). In Studies 2 and 3, we additionally manipulated the method of lie detection (direct vs. indirect). In contrast to the original hypotheses, we found that power led to increased veracity assessment accuracy. Having power over others enhances the accuracy of one’s veracity assessment, although this increase is small and limited to lie detection (Study 1) or direct judgments (Studies 2 & 3). Together, power affects the processing of social information and what aspects of this information are taken into account.

## Introduction

Lying is common in everyday interactions ([1,2], but see [3]). Thus, lie detection plays an important role in many contexts, from law enforcement [4] and jury verdicts [5] to business negotiations [6] and job interviews [7]. Specifically, as lying during recruitment is pervasive, has detrimental consequences for future job performance, and is associated with workplace deviance, veracity assessment is of paramount importance in the workplace context. Empirical research has revealed the dynamics behind everyday deception: in general, people are good at lying, and not so good at lie detection with a mean veracity assessment accuracy of around 54% [8,9]. Furthermore, it is usual to observe higher truth detection accuracy than lie detection accuracy, which stems from truth bias, that is, a tendency to judge messages as truths rather than as lies [10]. Past research has also repeatedly shown that people usually hold incorrect beliefs about cues to deception [11–15], thereby hinting at reliance on stereotypical behavioral cues when assessing the veracity of others as the reason behind poor lie detection [14,16–20].

Low deception detection accuracy and flawed knowledge of cues to deception have attracted a lot of empirical attention, resulting in a significant body of research. Some studies focused on procedural factors that influence veracity assessment, revealing improvement after participation in different lie detection training programs [21] or through the use of specific methods of interviewing [22,23]. Another line of research considered the role of personality or gender; however, the results did not bring conclusive patterns [8,9,24]. Finally, the role of social factors in veracity assessment accuracy was also tested, showing, for example, no significant differences in lie-truth judgments between professionals whose occupation requires credibility decisions and laypeople [8,14,17,25–27]. These studies, however, rarely investigated psychological dynamics in social interactions, such as those stemming from the hierarchical dimension of social relations as potential factors influencing one’s ability to detect lies. Social power differentiation may play a role in shaping the accuracy of lie-truth judgments.

### Social power

Power is commonly defined as asymmetric control over valued resources in social relationships [28], which can be exercised by providing or withholding resources or administering punishments [29]. Power dynamics affect a wide range of social interactions in personal [30], peer [31], and professional contexts [32,33]. Sadly, only too often, this influence results in socially undesirable effects. Having power promotes cheating and dishonesty [30,34,35]. Power was found to decrease empathic accuracy [36] and the ability to take the perspective of others [37], whereas it has been shown to increase confidence in one’s own judgments while actually decreasing the accuracy of the same [38].

Crucially, power increases reliance on easily accessible categorical information in person perception, thereby intensifying stereotyping ([39], but see [40]) and the tendency to seek less diagnostic information about others [41]. Moreover, in comparison to powerless individuals, powerholders focus more strongly on stereotype-consistent information, and they are guided, to a higher extent, by schematic knowledge and information associated with their expectancies [42]. To put it in a nutshell, power increases reliance on constructs that easily come to mind. In turn, this has the potential to diminish the ability of the powerful to detect lies because “a typical liar’s behavior” stereotype is strong and universal [13]. Thus, it can be hypothesized that power may increase the attention paid to stereotypic information about liars’ behavior and, at the same time, reduce the motivation to form an accurate perception of others. Hence, powerholders may be expected to be less accurate in terms of differentiating between those who lie and those who tell the truth as compared to those who do not hold power. Here, we aim to integrate the literature on social power with conclusions reached about lie detection.

### Overview of the studies

Drawing from the reviewed literature, we aimed to test how power is associated with lie detection accuracy. It is predicted that social power will have a negative effect on the ability to detect lies due to higher reliance on stereotypical cues. Across three experimental studies, we thus manipulated whether the senders lied or told the truth and asked participants to assess the senders’ veracity. Subsequently, we compared the responses of those in high power positions with a control group. In Study 1, we compared the responses of employees in managerial and non-managerial positions, and in Studies 2 and 3, we manipulated power, thus aiming to maximize both the external and internal validity of our work. For each study, we report how we determined our sample size, all the data exclusions (if any), all the manipulations, and all the measures. Data for all three studies are available at the Open Science Framework: https://doi.org/10.17605/OSF.IO/4R3VU

## Study 1

In Study 1, we aimed to test veracity assessment accuracy in a natural context. To this end, we recruited employees occupying managerial and non-managerial positions. We measured the participants’ subjective power as well as their knowledge of cues to deception in order to control for potential differences between those in non-managerial and managerial positions.

### Materials and methods

#### Participants

The participants were employees occupying managerial and non-managerial organizational positions in various sized organizations. We originally aimed to include 50 participants “per cell,” following the suggestion of [43]; however, we managed to recruit 44 managers, and consequently, we recruited an equal-sized group of non-managers, matched in terms of age, *t*(86) = 0.85, *p* = 0.398, *d* = 0.18; gender (50% women in both groups); years of occupational experience, *t*(86) = 1.23, *p* = 0.221, *d* = 0.27; and education (all the participants had a university degree). The final sample consisted of 88 employees aged between 26 and 64 (*M* = 43.57, *SD* = 10.02).

#### Materials

As a first step, we prepared a range of short video clips featuring mock job interviews, which we then used in all the studies presented here. Twenty undergraduate students from various universities (*M*_age_ = 22.40, *SD*_age_ = 3.13, 11 women) were recruited via social media advertisements to take part in a lie detection procedure. They were informed that their statements would be video recorded for scientific purposes, and they received a gift card worth approximately $8 as compensation. They were also informed they would receive an additional $35 gift card if they were judged by the experimenters to be among the most credible participants. As we aimed to prepare materials that would resemble real-life job interviews, we decided to use job positions of the senders’ choice as they would have been familiar with the typical requirements and qualifications for such positions. Thus, prior to the session, the senders were asked what positions they would apply for in a real-life situation and were asked to prepare and bring to the session their complete and accurate resumes, which described their work experience and education history, as it was necessary to establish ground truth. Moreover, they were asked to dress in clothing that was appropriate for an actual job interview.

During the recording session, the senders were asked by the experimenters’ confederate to imagine that they were applying for a position of their choice and were taking part in a recruitment interview. Then, they were asked to prepare one truthful and one deceptive presentation of their work experience, competence, and education with regard to the skills that were thought to be necessary for this position. The order of truthful and deceptive presentations was counterbalanced, and the senders were given 2 minutes to prepare a believable free narrative self-presentation before each recording. The videos showed the senders’ entire bodies from head to foot.

In order to check the senders’ self-assessment in terms of lying behavior, prior to recording they were asked a series of questions. Using 7-point Likert scales (1 = *definitely not*, 7 = *definitely* yes), they assessed the extent to which they perceived themselves to be good liars (*M* = 4.80, *SD* = 1.32, *t*(19) = 4.40, *p* < .001, *d* = 0.98) and how stressed (*M* = 3.85, *SD* = 1.56, *t*(19) = 1.0, *p* = .330, *d* = 0.22) and cognitively taxed (*M* = 3.90, *SD* = 1.52, *t*(19) = 1.18, *p* = .253, *d* = 0.26) they usually felt when they had been lying. Similar questions concerning the present lying situation were asked after the recordings. We then tested their answers against the mid-point of the scales (*i*.*e*., 3.5). The senders assessed their success at lying convincingly as being above the mid-point of the scale (*M* = 4.30, *SD* = 1.62, *t*(19) = 2.20, *p* = .040, *d* = 0.49). Furthermore, they were rather stressed (*M* = 4.60, *SD* = 1.46, *t*(19) = 3.36, *p* = .003, *d* = 0.75) and cognitively taxed (*M* = 4.30, *SD* = 1.22, *t*(19) = 2.94, *p* = .008, *d* = 0.66) when lying. The senders felt motivated to perform the task well the whole time (*M* = 5.30, *SD* = 1.17, *t*(19) = 6.85, *p* < .001, *d* = 1.53), and they somewhat cared about the opportunity to earn an additional gift card reward (*M* = 4.40, *SD* = 1.96, *t*(19) = 2.06, *p* = .054, *d* = 0.46).

Overall, this procedure allowed us to prepare two sets of videos that each contained 12 video clips: 6 featuring truthful senders and 6 featuring lying senders. The categorization of video clips as truthful or deceitful was additionally checked using the resume provided by each sender as the criterion for following the instructions. Each sender appeared only once within each set (with a deceptive or a truthful statement). The sets were balanced regarding the gender of the senders. Each participant in the main experiment was presented with one set of clips. The number of truthful and/or deceptive presentations was unknown to the participants. The length of the video clips varied between 53 seconds and 3 minutes and 5 seconds. In both sets, the videos were the same length (measured in seconds): *M* = 88.58, *SD* = 36.51 and *M* = 90.58, *SD* = 27.92 for sets 1 and 2, respectively, *t*(22) = -0.15, *p* = .882, *d* = -.06. The length did not differ with respect to gender: *M* = 80.67, *SD* = 24.71 and *M* = 98.50, *SD* = 36.48 for female and male senders, respectively, *t*(22) = 1.40, *p* = .175, *d* = .60.

#### Measures

*Subjective power*. This was measured with a four-item questionnaire (33), asking, for example, “To what extent do you have influence over people in your organization?” Participants were asked to respond on 7-point Likert scales ranging from 1 = *very little* to 7 = *a lot*, with higher scores indicating greater power over others (*M* = 3.28, *SD* = 1.71, α = .92).

*General confidence in one’s own ability to detect lies*. This was measured with two items: “Please rate your confidence in terms of your ability to detect lies” and “Please rate your confidence in terms of your knowledge regarding the cues used to detect lies.” Participants were asked to respond on 7-point Likert scales from 1 = *not at all confident* to 7 = *very confident*, with higher scores indicating greater confidence (*M* = 4.20, *SD* = 1.41, α = .92).

*Perception of the candidates*. The participants responded with their opinions as to whether or not the candidates were lying or telling the truth and to what extent they were certain of their judgments on a 7-point Likert scale ranging from 1 = *definitely not certain* to 7 = *definitely certain*. As each participant assessed both truthful and deceptive statements, two mean rates of correct classifications were computed: the truth detection accuracy rate and the lie detection accuracy rate. The rates were calculated by the assignment of a score of 1 when a truth (lie) was correctly identified or the assignment of a score of 0 when a judgment was incorrect. Then, the scores for truthful (lying) senders were added up and divided by the number of truthful (lying) senders (*i*.*e*., six). Both rates could range between 0 and 1.

*Knowledge of cues to deception*. This was measured with an inventory developed by Ulatowska [14] based on Akehurst et al.’s questionnaire [44] and DePaulo et al.’s meta-analysis of studies on behavioral cues to deception [45]. The participants were asked to assess 33 verbal and nonverbal behavioral cues with regard to how strongly, in their opinions, these cues are associated with deceptive behavior. The participants evaluated each cue on a 7-point Likert scale ranging from -3 to 3, where negative values denoted higher frequency, increased likelihood, or a stronger relationship between this cue and telling the truth; the mid-point of the scale (value 0) denoted “no differences between liars and truth-tellers”; and positive values denoted higher frequency, increased likelihood, or a stronger relationship with lying. Each response was then categorized as correct (1) or incorrect (0) based on the findings of DePaulo et al. [45]. The mean accuracy rate was calculated by adding the scores and dividing the sum by the number of cues; thus, it could range between 0 and 1, and a rate of .33 could be achieved by guessing [14].

#### Procedure

First, the participants responded to questions regarding their position, age, and gender and their subjective workplace power, as well as their general confidence in their ability to detect lies. Then, they were asked to carefully watch one set of 12 video clips and indicate whether each of the candidates was lying or not. Also, for each candidate viewed, participants were asked to assess their confidence in their judgment. Finally, we measured the participants’ knowledge of cues to deception.

### Results and discussion

#### Subjective power

As expected, individuals in high-power organizational positions reported having significantly more power (*M* = 4.49, *SD* = 1.41) than those in low-power positions (*M* = 2.06, *SD* = 0.94), *t*(86) = 9.53, *p* < .001, *d* = 2.06.

#### Perception of the candidates

A 2 (organizational position: high vs. low) x 2 (statement veracity: truthful vs. deceptive) mixed design ANOVA was utilized to test the effect of the power position on the accuracy of the veracity assessment. Organizational position was a between-participants factor, and veracity was a within-participants factor. A Bonferroni correction was applied to all simple effect analyses.

The main effect of veracity was significant, *F*(1, 86) = 30.11, *p* < .001, η^2^ = .26. In line with past work, truths were easier to detect (*M* = 0.60, *SD* = 0.22) than lies (*M* = 0.42, *SD* = 0.21). Although the main effect of organizational position was not significant, *F*(1, 86) = 2.45, *p* = .121, η^2^ = .03, the veracity and position interaction was significant, *F*(1, 86) = 9.14, *p* = .003, η^2^ = .10. A simple effect analysis revealed that individuals in higher-power positions were significantly better at lie detection (*M* = 0.49, *SD* = 0.22; *p* < .001) than individuals in lower-power positions (*M* = 0.34, *SD* = 0.17). However, there was no significant difference in the detection of truths between those in high- (*M* = 0.58, *SD* = 0.20) and low- (*M* = 0.63, *SD* = 0.23; *p* = .223) power positions. The veracity effect, that is, higher accuracy in terms of truth detection than lie detection [10], was observed among participants in lower positions (*p* < .001) but not among those in higher ones (*p* = .085). Furthermore, in the case of the latter group, the truth detection accuracy, *t*(43) = 2.53, *p* = .015, *d* = 0.63, but not the lie detection accuracy, *t*(43) = -0.22, *p* = .824, *d* = 0.03, was significantly greater than the chance-level accuracy (*i*.*e*., 0.5). In the low-power position group, both accuracy rates differed significantly from that of chance-level accuracy (truth: *t*(43) = 3.75, *p* = .001, *d* = 0.57; lie: *t*(43) = -5.93, *p* < .001, *d* = 0.89).

To test whether the veracity effect was the consequence of a tendency to judge more messages as truths than lies [46], truth bias was measured as the proportion of total messages judged as truthful. A group comparison showed that the truth bias was significantly higher, *t*(86) = 3.02, *p* = .003, *d* = 0.65, among individuals in lower-power positions (*M* = 0.64, *SD* = 0.14) than among those in higher-power ones (*M* = 0.54, *SD* = 0.17). Furthermore, the proportion of judgments of truthfulness was significantly above the level of chance among those in lower organizational positions, *t*(43) = 6.73, *p* < .001, *d* = 1.01, but not among those in higher positions, *t*(43) = 1.59, *p* = .119, *d* = 0.24.

#### Confidence

Two types of confidence judgments were compared between groups of participants occupying different positions in the organizational hierarchy. As expected, having a high-power position in an organization was related to increased general confidence in one’s ability to detect lies, *t*(86) = 3.26, *p* = .002, *d* = 0.70 (higher positions: *M* = 4.67, *SD* = 1.27; lower positions: *M* = 3.74, *SD* = 1.40), and in judgments of the senders’ veracity, *t*(86) = 3.86, *p* < .001, *d* = 0.83 (higher positions: *M* = 5.21, *SD* = 0.86; lower positions: *M* = 4.43, *SD* = 1.02).

#### Knowledge of cues to deception

Although the accuracy of the knowledge of deception cues was rather low in both groups, the rate for the high-power individuals (*M* = 0.36, *SD* = 0.06), *t*(43) = 3.55, *p* < .001, *d* = 0.53, exceeded the chance level (*i*.*e*., 0.33), albeit the effect being small in magnitude, whereas this was not the case for the low-power participants (*M* = 0.35, *SD* = 0.08), *t*(43) = 1.84, *p* = .073, *d* = 0.28. Furthermore, the difference between the groups was not significant, *t*(86) = 0.87, *p* = .387, *d* = 0.19.

#### Bivariate relations

Zero-order correlations are presented in Table 1. Perceived power over others was significantly positively correlated with general confidence in the ability to detect lies, as well as with confidence in veracity judgments and lie detection accuracy. Consequently, the latter correlation was accompanied by a significant negative correlation between power and truth bias–participants who had higher power were less prone to assess senders as truthful regardless of their actual veracity.

**Table 1:** Correlations between variables with 95% confidence intervals (Study 1).

Variable	1	2	3	4	5	6	7
1. Power over others	-						
2. General confidence in the ability to detect lies	.53*** [.37, .67]	-					
3. Overall detection accuracy	.10 [-.11, .30]	.04 [-.17, .25]	-				
4. Lie detection accuracy	.34** [.14, .51]	.19^+^ [-.02, .38]	.63*** [.48, .74]	-			
5. Truth detection accuracy	-.21^+^ [-.40, .002]	-.13 [-.33, .08]	.65*** [.51, .76]	-.19 [-.38, .02]	-		
6. Judgment confidence	.47*** [.29, .62]	.31** [.10, .49]	.15 [-.06, .35]	.32** [.12, .50]	-.12 [-.32, .09]	-	
7. Truth bias	-.36*** [-.53, -.16]	-.21^+^ [-.40, .003]	.02 [-.19, .23]	-.76*** [-.84, -.66]	.78*** [.68, .85]	-.29** [-.47, -.08]	-
8. Knowledge of cues to detect lies	.03 [-.18, .24]	-.15 [-.35, .06]	-.29** [-.47, -.09]	-.14 [-.34, .07]	-.23* [-.42, -.02]	.02 [-.19, .23]	-.06 [-.27, .15]

^+^ *p* < .10.

*p < .05.

**p < .01.

***p < .001.

The results of this field study, contrary to our initial hypotheses, revealed that participants who, objectively and subjectively, had more power, were more accurate than participants in lower positions in terms of performing lie detection (although not in truth detection). Notably, those in higher-power positions did not differ in their stereotypical beliefs about cues to deception from those in lower-power positions. Also, although lie detection accuracy was significantly higher among the high-power group, it was not different from that of the chance level (.50). Thus, this pattern implies that individuals who hold managerial positions might be more suspicious of job candidates as a result of years of professional experience [47]. In turn, this suspiciousness may be behind the observed differences between those in high- and low-power positions.

Moreover, powerholders’ lie detection accuracy might be a consequence of more flexible judgment strategies, resulting in them applying a different set of deception cues to different senders [48], paying increased attention to goals, and enhancing their focus on task-relevant details [49]. Previous studies also suggested that having power enhances attention and affects basic cognitive processes [50]. Thus, as suggested by the significant difference in terms of the knowledge of cues to deception between the chance level and those in higher-power positions, power might actually improve lie detection by increasing the focus on diagnostic cues to deception and by increasing the flexibility of the decision rules applied when analyzing the behavior of suspected individuals [48]. This beneficial role of power may be visible despite the rather stereotypical views on cues to deception (which, however, were still more accurate than mere guessing) explicitly declared by the high-power participants. A previous analysis showed that our beliefs about behaviors that accompany deception may actually be more accurate when we are applying them when making veracity judgments than when we are asked to merely report them [19]. Finally, although the participants in Study 1 were real-life powerholders, they did not have any power over the senders they were assessing, which might have influenced the accuracy of their veracity assessments. Thus, we designed Study 2 to overcome such limitations and verify the findings of Study 1.

## Study 2

In Study 2, we manipulated power by means of a role-playing task and asked participants to imagine being a Human Relations (HR) manager and having to decide which candidate is most trustworthy and hirable. Furthermore, Study 1 revealed that participants’ declarative beliefs about cues to deception are rather stereotypical, but still, the powerful group showed higher lie detection, but not truth detection, accuracy. This result might be caused by an enhanced focus on task-relevant details (*i*.*e*., diagnostic cues to deception) when the participants’ task was to detect lies. If that is the case, then this enhanced focus on task-relevant details should result in higher detection accuracy among the powerful when the task is explicitly described as being about lie detection. However, when the task is specified as being about social perception, as in the case of so-called indirect methods of lie detection, power should not lead to increased accuracy (and perhaps even result in decreased accuracy). Thus, in order to verify this prediction, we also manipulated the method of lie detection in Study 2.

A typically used lie detection procedure involves a direct truth-lie decision. However, as previously mentioned, when attempting to distinguish between lies and truths, people tend to focus on stereotypical rather than actual indicators of lying [11]. In order to minimize the risk of the activation of a stereotype of a liar, an indirect method of lie detection can be applied (for a review, see [51,52]). According to this approach, observers are asked to assess their own emotions or cognitions when receiving senders’ statements [53–55] or analyze characteristics of the senders’ behavior (*e*.*g*., [56,57]) rather than directly assessing the senders’ veracity.

Past research employed a broad range of indirect measures (for a review and discussion, see [52]). This may stand behind the inconclusive results observed in a recent meta-analysis, which revealed how most measures classified as indirect were no more accurate than those used in direct lie detection [58]. However, many former measures were based on non-diagnostic cues, such as gaze behavior or nervousness [45]. In contrast, studies that utilized more general indirect questions (*e*.*g*., “Is he or she thinking hard?”) revealed more accurate differentiation between truthful and deceptive statements than the direct method [58]. This may have been because direct veracity judgments are often based on multiple cues, and many of them are only stereotypically related to deception [59,60], whilst some indirect questions draw the observers’ attention to more diagnostic cues [57,61] or constellations of cues [59].

Summing up, in Study 2, we aimed to manipulate both power and the method of lie detection. Based on the results of Study 1, we hypothesized that the high-power group would demonstrate a higher level of accuracy when distinguishing between truthful and deceptive statements and that this increased accuracy would be manifested when the direct method, rather than the indirect method, was applied [25].

### Materials and methods

#### Participants and design

A 2 (power: control vs. high power) x 2 (method of lie detection: direct vs. indirect) x 2 (veracity of candidates: truthful vs. deceptive) design was used. The first two factors were between-participants variables, while the last one was a within-participants factor.

We aimed to detect a medium-sized effect of η^2^_p_ = .05 with a power of 1-β = .90 [62]. To this end, we set an a priori sample size of 200. In reality though, participants were tested in small groups, each of which watched the same set of clips. We did not want to exclude any of the volunteers; thus, there were a few more participants per condition. The final sample consisted of 214 undergraduate students (171 women) aged between 19 and 49 (*M* = 22.50, *SD* = 5.01), who participated in the study in exchange for the opportunity to be entered into a lottery where they could win a gift card worth approximately 8 euros.

#### Materials

The same 2 sets of 12 videos were used as in Study 1.

#### Measures

*Perception of the candidates*. In the direct lie detection condition, participants responded as to whether they believed a candidate was lying and to what extent they were certain of their opinion on a 9-point Likert scale ranging from 1 = *definitely not* to 9 = *definitely*. Following the majority of indirect lie detection studies, we used multipoint rating scales to measure the difference in the assessment of liars and truth-tellers (for a discussion about different types of scales in lie detection studies, see [25,63]). The purpose of the application of a confidence judgment was twofold: First, we aimed to design the procedures used in both conditions as congruently as possible so that both groups would answer an equal number of questions. Second, previous studies showed that perceivers claim to be more confident when they are judging truthful messages than deceptive messages [64], and this question could serve as an additional within-participants indirect measure [51]. In the indirect lie detection conditions, participants responded as to whether a candidate had to think hard during her or his speech and whether she or he had given the impression of being a convincing speaker using the same scale. The “thinking hard” indirect question was chosen based on previous studies that suggested that lying was more cognitively demanding than truth-telling [58] and on those that revealed that this question accurately discerns between true and false statements (reviewed in [56–58]). The “convincing impression” question was chosen for being more HR lie-scenario-specific since prior studies suggested that such an indirect question may be more effective [56]. Furthermore, past work revealed that liars made less plausible, friendly, and cooperative impressions than truth-tellers [45,58], which could translate into liars being less convincing.

*Subjective power*. This was measured with a single item, “How much power did you experience during this task?” with a scale ranging from 1 = *no power at all* to 7 = *a lot of power* (*M* = 3.52, *SD* = 1.55).

#### Procedure

Participants in the high-power condition were asked to imagine putting themselves in the position of an HR manager making hiring decisions. Their task was to carefully watch 12 clips that featured candidates. Next, they were informed that after watching each clip they would be asked for their opinion regarding the candidate and that, at the end of the study, they would be asked to indicate the one person they trusted most and would be most likely to hire. In order to strengthen the manipulation, we informed them that they held the power in terms of this task and that the choice was theirs to make. In the control condition, participants were informed that this was a self-presentation study and that their task was to watch the clips and form opinions regarding the people presented in them.

Subsequently, all the participants watched one of the two sets of self-presentations previously used in Study 1. After watching each clip, the participants were asked for their opinions regarding the person they viewed. Dependent on the condition, the participants were either directly asked whether or not the candidates were lying and to what extent they were confident in their judgments (direct condition) or whether each person was a convincing speaker and whether she or he had to think hard during her or his presentation (indirect condition).

Finally, all the participants were asked how much power they felt they had during this task, and participants in the high-power condition were asked to indicate which candidate was the most trustworthy and hirable. In both sets of videos, there were senders who made a more favorable impression than others. In both cases, in the opinion of over 30% of the participants, these were truthful senders. However, in both video sets, the second most trustworthy senders were lying, and they gained over 17% of the votes. After indicating their age and gender, the participants were debriefed and thanked. Finally, they were entered into the gift card lottery, and those who won were rewarded.

### Results and discussion

#### Manipulation check

First, we tested whether the power manipulation was effective. To this end, a *t*-test was conducted that compared the subjective power experienced by those in the control and experimental groups. As expected, the participants who imagined themselves in the position of an HR manager experienced having significantly more power (*M* = 3.75, *SD* = 1.61) than the participants in the control group (*M* = 3.30, *SD* = 1.46), *t*(212) = -2.18, *p* = .03, *d* = 0.30.

#### Perception of the candidates

A 2 (power: control vs. high power) x 2 (method: direct vs. indirect) x 2 (veracity of statement: truthful vs. deceptive) mixed design ANOVA was used to assess the accuracy of deception detection. Method and perceived power were between-participants factors, and veracity was a within-participants factor. The analyses were conducted separately for two indirect questions related to cognitive load and making a convincing impression, both of which were compared to the direct question (see Table 2). The answers to the “convincing impression” question were re-coded in reverse order so that lower scores on the scale were related to truth-telling for all the questions (*i*.*e*., a sender was telling the truth, making less cognitive effort, and giving a more convincing impression). A Bonferroni correction was applied to all simple effect analyses.

**Table 2:** Mean scores (with accompanying SDs) for the indirect and direct conditions as a function of power and veracity (Study 2).

	Control condition			High-power condition			
	direct	indirect “thinking hard”	indirect “convincing”		direct	indirect “thinking hard”	indirect “convincing”
truths	4.27 (1.38)	4.84 (1.01)	4.92 (0.65)		3.70 (1.06)	4.61 (1.24)	4.83 (1.05)
lies	4.49 (0.97)	4.75 (1.07)	4.95 (0.88)		4.16 (1.30)	4.59 (1.19)	4.81 (0.97)

*Thinking hard indirect question vs*. *direct question*. The analysis revealed a marginally significant main effect of veracity, *F*(1, 210) = 3.49, *p* = .063, η^2^ = .02. As expected, liars were assessed as being more deceptive and having to think harder (*M* = 4.49, *SD* = 1.15) than truth-tellers (*M* = 4.34, *SD* = 1.26). There was also a main effect of perceived power, *F*(1, 210) = 5.32, *p* = .022, η^2^ = .02, which indicated that participants with high power assessed senders as being more truthful or having to think less hard (*M* = 4.27, *SD* = 1.16) than participants from the control condition (*M* = 4.57, *SD* = 0.94). To further test this rather unexpected result, we decided to check if these means differed significantly from 5, that is, the mid-point of the 9-point scale. Two one-sample *t*-tests found that raters in both the high-power condition, *t*(105) = -6.52, *p* < .001, *d* = 0.63, and the control condition, *t*(107) = -4.79, *p* < .001, *d* = 0.46, were biased toward making more truth/thinking-less-hard than lie/thinking-hard judgments.

There was also a significant main effect of method, *F*(1, 210) = 15.29, *p* < .001, η^2^ = .07, as well as an interaction effect of method and veracity, *F*(1, 210) = 6.59, *p* = .011, η^2^ = .03. Yet, the simple effect analysis revealed that for both types of lie detection methods, discernment between truthful and deceptive statements was only marginally significant (both *p*s = .11). Furthermore, only in the direct condition did we observe a pattern of results in accordance with our expectations; truth-tellers were assessed as being more truthful (*M* = 4.00, *SD* = 1.26) than liars (*M* = 4.33, *SD* = 1.14), whilst in the indirect condition, truth-tellers were assessed as having to think harder (*M* = 4.72, *SD* = 1.14) than liars (*M* = 4.67, *SD* = 1.13).

Neither veracity and power interaction, *F*(1, 210) = 0.99, *p* = .322, η^2^ = .005, nor the method and power interaction, *F*(1, 210) = 0.87, *p* = .353, η^2^ = .004, were significant. Although there was no significant three-way interaction between veracity, method, and power, *F*(1, 210) = 0.29, *p* = .589, η^2^ = .001, an exploratory simple effects analysis was conducted. As listed in Table 2, participants were only able to accurately distinguish between truthful and deceptive statements when a direct method of lie detection was utilized in the high-power condition (*p* = .003).

*Convincing impression indirect question vs*. *direct question*. A similar analysis was conducted for the second indirect question. There was a significant main effect of veracity, *F*(1, 210) = 4.37, *p =* .038, η^2^ = .02. Again, as expected, the ratings for liars (*M* = 4.59, *SD* = 1.08) were higher than those for truth-tellers (*M* = 4.42, *SD* = 1.18). Both the main effect of the method, *F*(1, 210) = 35.66, *p <* .001, η^2^ = .15, and the main effect of power, *F*(1, 210) = 5.48, *p =* .020, η^2^ = .025, were also significant. Once more, participants with high power assessed senders as more truthful or more convincing (*M* = 4.37, *SD* = 1.07) than participants from the control condition (*M* = 4.63, *SD* = 0.83). One-sample *t*-tests revealed that the average ratings of both participants with high power, *t*(105) = -6.04, *p* < .001, *d* = .59, and those from the control condition, *t*(107) = -4.63, *p* < .001, *d* = -.45, were significantly lower than five (the mid-point of the scale) and, thus, biased toward truth/more convincing judgments.

An interaction was observed between veracity and the method, *F*(1, 210) = 4.49, *p* = .035, η^2^ = .02. We did not repeat the analysis of the simple effects for veracity in the direct method condition as they were the same as in the previous model of comparison reported above. Once more, in the indirect method condition, participants did not distinguish between truthful and deceptive statements (*p* = .985). The interaction effects between veracity and power, *F*(1, 210) = 0.32, *p* = .571, η^2^ = .002, and between power and the method, *F*(1, 210) = 1.95, *p* = .164, η^2^ = .01, were not significant. Furthermore, an exploratory simple effect analysis was conducted for the non-significant three-way interaction of power, veracity, and method, *F*(1, 210) = 0.79, *p* = .375, η^2^ = .004. As presented in Table 2, the analysis revealed that the indirect question about being convincing did not differentiate accurately between truthful and deceptive statements.

An additional analysis was conducted to compare the two indirect questions within the indirect condition. It revealed that the “thinking hard” and “convincing impression” questions did not differ in terms of their accuracy in distinguishing between truths and lies (all *p*s > .1 in simple effects for question x veracity x power interaction).

#### Confidence judgments

Moreover, for the direct condition only, an additional 2 (power: control vs. high power) x 2 (veracity of statement: truthful vs. deceptive) mixed design ANOVA was conducted with confidence in the judgment as the dependent variable. Perceived power was a between-participants factor, while veracity was a within-participants factor. This ANOVA revealed a significant effect of power, *F*(1, 110) = 9.37, *p =* .003, η^2^ = .08. In line with past work [38], power increased confidence in one’s own judgments, as participants in the high-power condition were more confident in their judgments (*M* = 7.10, *SD* = 0.93) than those in the control condition (*M* = 6.41, *SD* = 1.40). Neither the main effect of veracity, *F*(1, 110) = 0.25, *p =* .620, η^2^ = .02, nor the power x veracity interaction effect, *F*(1, 110) = 1.78, *p =* .185, η^2^ = .02, was significant. Although participants from the high-power condition were, as expected, more confident about their veracity judgments, their confidence did not differ when they were judging truthful and deceptive candidates. The latter effect is contrary to previous studies that revealed higher confidence when judging truthful messages than deceptive messages [64]. However, this result confirms the limited accuracy of the indirect method applied using the “thinking hard” and “convincing impression” questions.

The results of Study 2 found that power improved veracity assessment, although this effect was only significant in the direct condition (*i*.*e*., when participants were openly asked whether each candidate was lying or telling the truth). The difference in the direct judgments of truthful and deceptive senders made by the powerful participants (0.46) was larger than the difference in the judgments made by the control group (0.23). However, when the participants were asked indirectly about their more general impressions of each candidate, we did not observe any differences in the assessment of truthful and lying senders. Although this seemingly contradicts some studies on indirect lie detection (*e*.*g*., [56,57,65]), other studies discovered that this method may be fallible (for a review, see [58]).

The increased accuracy of the direct veracity assessment in the powerful group, as compared to the control group, might suggest the higher suspiciousness of the former. However, the judgments in all the conditions did not exceed a value of 5, the mid-point of the scale. Significant bias toward truth [10] was, thus, visible regardless of senders’ veracity in both the power and the control conditions when a direct question was utilized (*p*s < .001). The judgment shift toward the truth was further revealed in the power condition when the “thinking hard” indirect question was asked–powerful judges were more inclined to assess both truthful and deceptive senders as not thinking hard (*p*s < .001). This bias was not significant (*p*s >.1) in the control condition or in either of the power conditions when a “convincing impression” indirect question was utilized.

Summing up, the results of Study 2 showed that power improves veracity assessment, though only when the task is explicitly formulated as such. Thus, it extends the results of Study 1. This pattern may support the conclusions of previous studies that suggest that power enhances the processing of information and leads to more flexible judgment strategies [50].

## Study 3

The aim of Study 3 was twofold. First, we aimed to replicate the effects of Study 2. Second, in order to increase theoretical and external validity, we sought to manipulate power differently.

### Materials and methods

#### Participants and design

Again, as in Study 2, we set an a priori sample size of 200. Participants were thus 200 students (181 women) aged between 19 and 26 (*M* = 22.02, *SD* = 1.58). They were told that they could take part in a lottery as a reward for participating where they could win a gift card worth approximately 8 euros. The design was the same as in Study 2.

#### Materials, procedure, and measures

In the power condition, participants were given control over resources they could offer as a reward to the candidate of their choice. They were informed that their task was to evaluate said candidates based on their job interviews and then choose the one who made the best impression and deserved to be rewarded or decline from picking anyone. In both video sets, similar senders to those in Study 2 were nominated to be rewarded by the largest group of participants. The participants then were given an instruction that stated that, in this task, they had power over others. Both the control condition and the rest of the materials and the procedure were identical to those in Study 2.

*Perception of the candidates*. This was measured as in Study 2.

*Subjective power*. This was measured as in Study 2 (*M =* 3.98, *SD =* 1.37).

### Results and discussion

#### Manipulation check

Again, we first tested whether power manipulation was effective. As expected, the participants who were given the resources and the choice of whether or not to reward any of the candidates experienced significantly more power (*M* = 4.31, *SD* = 1.24) than the participants in the control group (*M* = 3.66, *SD* = 1.42), *t*(196) = 3.41, *p* = .001, *d* = 0.49.

#### Perception of the candidates

A 2 (power: control vs. high power) x 2 (method: direct vs. indirect) x 2 (veracity of statement: truthful vs. deceptive) mixed design ANOVA was utilized to test the perceived power influence on the accuracy of the direct and indirect methods of deception detection. The methods and perceived power were between-participants factors, and veracity was a within-participants factor. As in Study 2, the analyses were conducted for two indirect questions separately with regard to the cognitive load and making a convincing impression, and the answers to some questions were re-coded in reverse order (see Table 3). A Bonferroni correction was applied to all the simple effect analyses.

**Table 3:** Mean scores (with accompanying SDs) for the indirect and direct conditions as a function of power and veracity (Study 3).

	Control condition			High-power condition			
	direct	indirect “thinking hard”	indirect “convincing”		direct	indirect “thinking hard”	indirect “convincing”
truths	4.04 (1.18)	5.43 (1.26)	4.49 (0.94)		3.42 (1.22)	5.21 (0.92)	4.73 (1.09)
lies	4.05 (1.16)	5.34 (1.09)	4.48 (0.95)		3.75 (1.38)	5.41 (1.16)	4.47 (1.12)

*Thinking hard indirect question vs*. *direct question*. As in Study 2, a marginally significant main effect of veracity was revealed, *F*(1, 196) = 2.82, *p* = .094, η^2^ = .01. As expected, liars were assessed as being more deceptive and as having to think harder (*M* = 4.64, *SD* = 1.41) than truth-tellers (*M* = 4.52, *SD* = 1.41). Also, a marginally significant main effect of power was found, *F*(1, 196) = 3.10, *p* = .080, η^2^ = .02. In line with Study 2, the high-power condition participants judged the senders as being more truthful or having to think less hard (*M* = 4.45, *SD* = 1.17) than the participants from the control condition (*M* = 4.71, *SD* = 1.17). A series of one-sample *t*-tests was conducted to determine whether participants with power were biased toward making more truth/thinking-less-hard than lie/thinking-hard judgments than the control group (*i*.*e*., if their ratings differed significantly from 5). This bias was expressed again in both the high-power condition, *t*(99) = -4.05, *p* < .001, *d* = .40, and the control condition, *t*(99) = -2.23, *p* = .028, *d* = 0.22.

The main effect of the method was significant, *F*(1, 196) = 101.21, *p* < .001, η^2^ = .34, as well as the interaction effect of power and veracity, *F*(1, 196) = 5.13, *p* = .025, η^2^ = .03. The simple effect analysis revealed that discernment between truthful and deceptive statements was only accurate (*p* = .006) in the high-power condition. Furthermore, neither the veracity and method interaction, *F*(1, 196) = 0.71, *p* = .402, η^2^ = .004; the power and method interaction, *F*(1, 196) = 1.58, *p* = .210, η^2^ = .01; nor the three-way interaction of method, power, and veracity, *F*(1, 196) = 0.35, *p* = .851, η^2^ = .00, was significant. However, in accordance with Study 2, a simple effects analysis for the latter interaction revealed that accurate discernment between truthful and deceptive statements was again observed only when the direct method of lie detection was utilized by the high-powered raters (*p* = .014).

*Convincing impression indirect question vs*. *direct question*. An analysis that compared truth-lie discrimination between the direct and indirect questions (whether a convincing impression was made) revealed a significant main effect of method, *F*(1, 196) = 25.50, *p* < .001, η^2^ = .12. However, the main effects of veracity, *F*(1, 196) = 0.08, *p* = .782, η^2^ < .001, and power, *F*(1, 196) = 1.42, *p* = .235, η^2^ = 0.007, as well as the veracity x power interaction, *F*(1, 196) = 0.08, *p* = .782, η^2^ < .001, were not significant. Nevertheless, it was again shown that participants in both the high-power condition, *t*(99) = -7.60, *p* < .001, *d* = 0.76, and in the control condition, *t*(99) = -7.53, *p* < .001, *d* = 0.75, were significantly biased toward truths and judgments of more convincing impressions. There was a significant interaction effect of method and power, *F*(1, 196) = 3.98, *p* = .048, η^2^ = .02. This effect was a consequence of the lack of differences between the high-power (*M* = 4.60, *SD* = 1.11) and control conditions (*M* = 4.49, *SD* = 0.94) in ratings of whether the senders made a convincing impression (*p* = .571). However, such a difference was visible in the ratings of veracity (*p* = .025; high-power condition: *M* = 3.58, *SD* = 1.30; control condition: *M* = 4.04, *SD* = 1.17).

The interaction of veracity and method was significant, *F*(1, 196) = 4.49, *p* = .035, η^2^ = .02. A simple effects analysis showed once more that the direct question distinguished more accurately (the difference approached significance, *p* = 0.09) between truthful (*M* = 3.73, *SD* = 1.20) and deceptive (*M* = 3.90, *SD* = 1.27) statements as compared to an indirect question (*p* = 0.194; truth: *M* = 4.61, *SD* = 1.02; lie: *M* = 4.48, *SD* = 1.03). The simple effects analysis for the three-way interaction of power, method, and veracity, *F*(1, 196) = 4.21, *p* = .041, η^2^ = .02, further clarified this result, and in agreement with Study 2, only the high-power group was responsible for the difference (*p* = .019).

An additional analysis comparing two indirect questions revealed that the “thinking hard” and “convincing impression” questions did not differ in terms of their accuracy of distinguishing between truths and lies (all *p*s > .05 in simple effects for the question x veracity x power interaction).

#### Confidence judgments

Additionally, for the direct condition only, the confidence ratings were compared. A 2 (power: control vs. high power) x 2 (veracity of statement: truthful vs. deceptive) mixed design ANOVA revealed that the main effects of veracity, *F*(1, 98) = 0.61, *p* = .437, η^2^ = .01 and power, *F*(1, 98) = 0.007, *p* = .931, η^2^ < .001, as well as the veracity x power interaction effect, *F*(1, 98) = 0.08, *p* = .931, η^2^ < .001, were not significant. Thus, as opposed to in Study 2, the participants in the high-power (*M* = 7.00, *SD* = 1.30) and control (*M* = 6.99, *SD* = 0.99) conditions did not differ in terms of their confidence judgments.

Study 3 replicated the main results of Study 2: power increased the accuracy of direct discernment between truthful and deceptive statements in the direct condition, though not in the indirect condition. Furthermore, regardless of a sender’s veracity, participants in the direct condition were significantly (*p*s < .001) biased toward truths (*i*.*e*., their judgments were significantly lower than the mid-point of the scale).

## General discussion

The results of the three studies converged and demonstrated how holding power over others affects lie detection. This was demonstrated for different operationalizations of social power: when subjective workplace power was measured (Study 1), when participants assumed a managerial role for the evaluation of job candidates (Study 2), and when they were given control over resources that they could choose whether to allocate to job candidates (Study 3).

The pattern of the results, however, contradicted our initial expectations that power undermines lie detection. Actually, in Study 1, participants occupying higher organizational positions were more accurate in terms of their detection of deceitful senders than participants in lower positions. Those in higher positions, however, were not more accurate with regard to the detection of truth. When we manipulated power (and the lie detection method) in Studies 2 and 3, we only observed more accurate discernment between truthful and deceptive statements among the high-power group when they were directly asked to judge the veracity of potential candidates. The powerful participants perceived candidates who were telling lies about their education and past work experience as significantly less truthful than those who told the truth about their career paths.

How can this pattern of results be explained? The increased accuracy of powerful participants in direct lie detection could be a consequence of them paying increased attention to goals [49], in this case, catching liars in a workplace context. Those in powerful positions may be more motivated to identify liars among potential candidates, as people who lie about their education and experience may prove incompetent [cf. 66], thereby putting powerholders’ own positions at stake. In line with this theorizing, we did not observe differences in the perception of the same candidates in other respects (*i*.*e*., in the indirect lie detection condition). Liars were perceived as equally convincing and as thinking as hard as those who were truthful, regardless of the condition. Although in other contexts indirect questions have been shown to improve lie detection (*e*.*g*., [57]), a meta-analysis showed that the accuracy of this method has varied across studies [58]. This previously observed lack of consistency may stem from the fact that many of the indirect questions were based on indicators that were not reliable cues to deception (*e*.*g*., eye contact, [58]) or from the inconsistency between the indirect questions and diagnostic cues presented by a liar [56,60]. This points to the necessity for further research on the improvement of indirect methods of lie detection. Nevertheless, this finding is in line with a decreased focus on others by the powerful [36,37] and their lower motivation in terms of achieving accurate perceptions of others [67], which may be behind the ineffectiveness of indirect methods of lie detection among such groups (though at the same time not impair their lie detection ability when using the direct method).

Furthermore, power enhances one’s ability to focus on task-relevant details [49] and, according to the situated focus theory of power, increases processing flexibility [50]. At the same time, it is suggested that successful lie detectors should consider both interpersonal and intrapersonal differences in the behavior of suspected individuals [48] and base their decisions on reliable, preferably multiple, cues [59,68]. It is possible then that when assessing veracity, powerful participants were able to concentrate on more diagnostic cues in senders’ behavior and apply more flexible decision rules. To confirm this hypothesis, future studies should directly test the strategies used by powerful lie detectors. Interestingly, we found divergent results between studies conducted in natural and laboratory contexts concerning truth bias. Namely, we observed truth bias in the judgments of those in non-managerial positions (Study 1) and the judgments of those in powerful positions (Studies 2 and 3). On the one hand, powerholders’ truth bias is not in line with motivated cognition accounts, predicting that powerless rather than powerful persons should be more trustful in order to avoid anxiety evoked by being dependent on others. In fact, in exchange relationships, individuals in higher-power positions trust others less than powerless people [69]. On the other hand, the bias toward truth is in line with meta-analyses indicating that laypeople judge messages of others as truthful rather than deceptive [8,25]. This judgmental bias depends on the availability heuristic [70]: in daily life, an average person is more often exposed to truthful statements than deceptive ones and, as a result, more likely to perceive others as truthful rather than deceptive [48]. Furthermore, the source of power in Studies 2 and 3 stemmed from a temporary role assigned during the experiment rather than in real life and often from years of experience in a managerial position. The lack of truth bias observed among those who actually occupied high-ranking organizational positions could be a consequence of frequent workplace deception. Studies have revealed that approximately 45% of employees admit to lying in the workplace [71] and that the most common lies told to superiors concern the protection of oneself, the protection of others, and getting time off. In the same vein, 24% of hiring managers admitted firing an employee for dishonesty [47]. Also, the lack of truth bias among individuals who are frequently exposed to deception is in line with studies on law-enforcement professionals (*e*.*g*., [72]) and prisoners (*e*.*g*., [73]), who reveal heightened levels of suspicion and a tendency to judge messages as deceptive rather than truthful (so-called *lie bias*). In fact, the mere monitoring of others’ performance levels results in lowered trust toward them [74]. Although we did not reveal lie bias in participants with higher organizational positions, their truth-lie judgments were not biased toward truth either. Thus, power affects how social information is processed and what aspects of social information are taken into account [66].

Moreover, in Study 1, which operationalized power as a high position within the organizational hierarchy, and in Study 2, we observed increased confidence among the powerful–a finding in line with past work (*e*.*g*., [75]). However, this increased confidence in Study 1 was related to lie detection accuracy but not to truth detection accuracy. Prior research also found that confidence is not highly related to accuracy in the domain of lie detection [8] or elsewhere [76,77]. In a similar vein, knowledge of cues to deception was not higher among the more confident and powerful, but it was negatively related to accuracy. This suggests that people in general, including those who hold power over others, may not be the best judges of their own abilities.

## Limitations and future directions

We know of no other study that has directly tested the effect of power on the accuracy of veracity assessment comparing direct and indirect methods of lie detection. Thus, it is important to further test this problem. Future studies should check the effect of social power in contexts other than job applications and check the lie detection accuracy of real-life powerholders when they have actual power over the individuals they are evaluating. One may argue that there are many lie detection studies with participants who are considered to be powerful (*e*.*g*., law-enforcement employees). Their subjective power, however, has not often been measured. Moreover, some of our unpublished data collected from customs officers suggest that members of such groups may not consider themselves as more powerful than participants from control groups.

Furthermore, although Study 1 tested declarative beliefs about cues to deception, future studies should compare the cues actually used by high- and low-power individuals when assessing others and compare them to cues displayed by senders (see [19]). Finally, it would be worthwhile to investigate lie detection among those who are subjected to the power of senders.

## Concluding remarks

Employee dishonesty has negative consequences for both co-workers and organizations as immoral acts can increase the cost of monitoring workers, reduce job performance and the reputation of a company, as well as increase stress and decrease job satisfaction in fellow employees [78]. Undetected lies may have even more serious consequences in other domains (*e*.*g*., in the legal context). In line with past work, we observed that powerful people’s beliefs about behavior related to deception were, in general, stereotypical (Study 1), although at the same time, the powerful displayed higher levels of confidence in terms of both their own abilities to detect lies and their knowledge of cues to deception, as well as with regard to the accuracy of their specific truth-lie judgments (Studies 1 & 2). Importantly, however, we also found that power over others increases the accuracy of one’s veracity assessments, even though this increase is small and limited to lie detection (Study 1) or direct judgments (Studies 2 & 3). In light of the broad, negative consequences of deception, individuals in positions of power should aim to be more accurate when performing lie detection. We found that having power is, to some extent, conducive to that goal. Powerholders, however, should also be aware of their limited knowledge and abilities in terms of veracity assessment. Their increased confidence accompanied by rather limited lie detection skills, which were both observed here, may have grave consequences in workplace, political, and legal contexts.
